# HDL-C to hsCRP ratio is associated with left ventricular diastolic function in absence of significant coronary atherosclerosis

**DOI:** 10.1186/s12944-019-1157-6

**Published:** 2019-12-12

**Authors:** Lufan Sun, Xiaorui Liu, Wenna Li, Dalin Jia

**Affiliations:** 1 Department of Cardiology, The First Hospital of China Medical University, 155 North Nanjing Street, Shenyang, 110001 China; 2 Intensive Care Unit, People’s Hospital of Huanren County, Benxi, China

**Keywords:** Left ventricular diastolic function, High-density lipoprotein cholesterol, Inflammation, Cardiovascular risk factor

## Abstract

**Background:**

High-density lipoprotein cholesterol (HDL-C) is considered as a protective marker of coronary atherosclerotic disease (CAD). It is still not clear if HDL-C is associated with left ventricular (LV) diastolic function in an inflammation-related manner in absence of significant coronary atherosclerosis.

**Methods:**

392 patients who complained of chest pain and were suspected of CAD without heart failure were enrolled in this study. Coronary angiography or coronary artery CT scan was performed to detect coronary atherosclerosis. Transthoracic echocardiography was performed to evaluate cardiac function. Plasma level of HDL-C and high-sensitive C-reactive protein (hsCRP) were determined in each subject. Relationship between HDL-C/hsCRP ratio and LV diastolic function in subjects without significant coronary atherosclerosis was investigated.

**Results:**

204 subjects without significant coronary plaques were analyzed finally, including 84 males and 120 females whose ages ranged from 30 to 84 years old. When divided into HDL-C/hsCRP quartiles, those in the fourth quartile demonstrated the best diastolic function (E/*e’* 10.14 ± 2.87, *P* = 0.02 ). HDL-C/hsCRP was the most significant factor correlated with E/*e’* in univariate regression analysis (*r* = − 0.232, *P* < 0.001) and multiple regression analysis adjusted by other factors (standardized β = − 0.258 , *P* < 0.0005 ). In logistic regression, HDL-C/hsCRP was proved to be a protective factor of LV diastolic dysfunction E/*e’* > 14 (OR = 0.649, 95%CI 0.444–0.948,*P* = 0.025 ). The sensitivity and specificity of using HDL-C/hsCRP < 0.98 to predict LV diastolic dysfunction were 64.3% and 56.2%, respectively. HDL-C/hsCRP ratio presented a reduced trend as increasing rate of CV risk factors.

**Conclusions:**

HDL-C/hsCRP ratio strongly correlates with LV diastolic function in absence of significant coronary atherosclerosis. Low HDL-C/hsCRP ratio tends to relate with LV diastolic dysfunction.

## Background

Reduced left ventricular (LV) diastolic function is the base of heart failure with preserved ejection fraction (HFpEF), a clinical diagnosis defined as symptomatic heart failure with an ejection fraction (EF) ≥50% [1]. HFpEF makes up half of heart failure cases and prevails over heart failure with reduced ejection fraction (HFrEF) especially in old people [2, 3]. The population of LV diastolic dysfunction without the symptoms of congestive heart failure such as dyspnea is astonishing but insidious as well [4]. Early detection of this imperceptible situation may help decrease the possibility of progressive heart failure [5].

More and more researches indicate that diastolic function decrease mainly originates from cardiovascular (CV) risk factors-related chronic inflammation other than cardiomyocytes loss, which usually causes systolic dysfunction [6]. People with known CV risk factors, such as hypertension, diabetes, obese, hypercholesterolemia and smoking are in a state of elevated inflammation of the heart and susceptible to diastolic function impairment [7–9]. Compared with acute infection, the chronic inflammatory status following CV risk factors requires a more sensitive assay. High-sensitive C-reactive protein (hsCRP) is a well-established inflammation marker, which can accurately detect very low levels of CRP even in healthy individuals. It is valuable to predict cardiovascular disease morbidity and treatment effects [10].

High-density lipoprotein cholesterol (HDL-C) has been considered as a protective marker of cardiovascular disease for decades [11]. Framingham Research demonstrated that low HDL-C was related to morbidity of both coronary heart disease and heart failure [12, 13]. Formation of atherosclerosis is attributed to excess cholesterol and inflammation [14, 15], while HDL plays a scavenger role removing deposited cholesterol from macrophages and relieves inflammation [16, 17]. Myocardial ischemia caused by significant coronary atherosclerosis is one of the reasons for reduced LV diastolic function even before systolic function impairment [18]. However, to date, how HDL-C relates with LV diastolic function of a heart without obvious coronary atherosclerosis has not been fully elucidated yet. We hypothesize that HDL-C is favorably associated with LV diastolic function in an inflammation-related manner and the combination of HDL-C and hsCRP may be useful to estimate LV diastolic function. Therefore, we aim to investigate the relationship between HDL-C/hsCRP ratio and LV diastolic function in subjects without significant coronary atherosclerosis in this study.

## Methods

### Study population

Three hundred and ninety-two patients who complained of chest pain and were suspected of coronary atherosclerotic disease (CAD) were recruited as participants from Department of Cardiology in the First Hospital of China Medical University during January 2017 to May 2017. The study proceeded under the approval of ethics committee of this hospital in accordance with Declaration of Helsinki. Written informed consents were obtained from all the subjects at the beginning. Those who had atrial fibrillation episodes or history, acute infection or were diagnosed chronic inflammatory diseases were excluded in order to avoid bias of results. Patients diagnosed as heart failure previously and currently were not included either. All participants were free of definite CAD including myocardial infarction history, previous percutaneous coronary intervention or coronary artery bypass grafting. At least one of the tests including ambulatory ECG, exercise ECG and stress myocardial perfusion by SPECT was performed in addition to resting ECG when necessary in all the participants to detect significant myocardial ischemia. Coronary angiography or coronary artery CT scan were finally performed in those subjects without detectable myocardial ischemia following a standard procedure to make clear whether they had obvious coronary atherosclerotic lesion with≥50% diameter stenosis.

CV risk factors including current smoking, hypertension, diabetes, obese and hypercholesterolemia, were recorded. Besides previous confirmed diagnosis and ongoing treatment, patients were evaluated as follows. Blood pressure was determined using conventional cuff method and hypertension was defined as systolic blood pressure (SBp) ≥140 mmHg and/or diastolic blood pressure (DBp) ≥90 mmHg according to the WHO guideline at that time. Diabetes was defined as fasting plasma glucose (FPG) ≥7.0 mmol/L and/or hemoglobin A1C (HbA1C) ≥6.5% according to 2010 American Diabetes Association criteria. Body mass index (BMI) was calculated as the body weight (in kilogram) divided by square of height (in meter). Obese was defined as BMI ≥ 28 kg/m^2^ according to Chinese guideline. Hypercholesterolemia was defined as plasma level of total cholesterol (TC) > 5.72 mmol/L with or without plasma level of low-density lipoprotein cholesterol (LDL-C) > 3.64 mmol/L in a fasting state.

### Laboratory measurements

Blood samples were collected after at least 8 h overnight fasting. TC, LDL-C, FPG, HbA1C, plasma triglyceride (TG), high-density lipoprotein cholesterol (HDL-C), uric acid (UA) and cystatin C (Cys C) were measured by standard method in the clinical laboratory of the same hospital. Plasma hsCRP was analyzed by immunophelometry method using Siemens hsCRP immumoreagent on a Siemens BNII instrument.

### Cardiac function evaluation

Transthoracic echocardiography was performed using Vivid 7 GE system. We firstly measured left atrial diameter (LA), LV end-diastolic diameter and the thickness of both LV septal and posterior walls by M-mode echocardiography and two-dimensional echocardiography. Left ventricular ejection fraction (LVEF) was obtained using biplane Simpson’s methods from the apical 2- and 4- chamber views. Peak mitral flow velocity in early diastole (E) and late diastole (A) were measured from apical 4-chamber view and the ratio of E/A was calculated. We further performed pulse-wave tissue Doppler echocardiography. Peak early diastolic velocity of mitral annular was measured in both septal and lateral parts. Mean mitral annular early diastolic velocity *e’* was calculated from their average. E/*e’* was calculated as a parameter of LV diastolic function with the other parameters LA, E/A and *e’*. According to the 2016 American Society of Echocardiography criteria [19], E/*e’* > 14 was considered as diastolic dysfunction. All the ultrasound data were sampled from 3 cardiac cycles and averaged.

### Statistical analysis

Statistical analysis was performed using SPSS 22.0 software (SPSS Inc., Chicago, IL, USA). Continuous variables were expressed as means ± standard deviation (SD) while categorical variables were expressed as counts or percentage. Continuous variables were compared using analysis of variance (ANOVA) among groups followed by S-N-K test. Chi-square test or Fisher’s exact test was performed to determine if categorical variables were different across groups. Pearson analysis and Spearman analysis were used to evaluate univariate correlations for continuous variables and categorical variables respectively. Stepwise multiple regression analysis was also performed to confirm independent determinants. Logistic regression analysis with odd ratio (OR) and 95% confidence interval (CI) were performed to estimate possible association between parameters and diastolic dysfunction. *P* < 0.05 was considered to be statistically significant.

## Results

### General characteristics of subjects

Two hundred and four patients were enrolled in final analysis after excluding those with obvious coronary plaques according to the results of coronary angiography or coronary artery CT scan. Among these subjects, there were 84 males and 120 females and the age ranged from 30 to 84 years old with an average of 60.2 ± 10.2 years old. Clinical and echocardiographic characteristics of these subjects were shown in Table 1.

**Table 1:** General clinical and echocardiographic characteristics of the subjects

Male gender (n /%)	84/41.2
Age (years)	60.2 ± 10.2
Current smoker (n /%)	59/28.9
Hypertension (n /%)	133/65.2
Diabetes mellitus (n /%)	38/18.6
Obese (n /%)	38/18.6
Hypercholesterolemia (n /%)	52/25.5
Statin use (n /%)	163/79.9
Other lipid regulator use (n /%)	30/14.7
ACE inhibitor use (n /%)	92/45.1
β blocker use (n /%)	103/50.5
Heart rate (beats/min)	78.1 ± 13.4
Systolic blood pressure (mmHg)	141.8 ± 22.8
Diastolic blood pressure (mmHg)	83.1 ± 15.3
Body mass index (kg/m^2^)	25.2 ± 2.9
Fasting plasma glucose (mmol/L)	5.89 ± 1.88
Hemoglobin A1C (%)	6.09 ± 1.11
Total cholesterol (mmol/L)	4.75 ± 1.05
Low-density lipoprotein cholesterol (mmol/L)	3.03 ± 0.89
High-density lipoprotein cholesterol (mmol/L)	1.14 ± 0.29
Triglyceride (mmol/L)	1.78 ± 1.42
Uric acid (umol/L)	309.3 ± 77.4
Cystatin C (mg/L)	0.83 ± 0.19
High-sensitive C-reactive protein (mg/L)	1.54 ± 1.60
LA (mm)	35.5 ± 3.8
LVDD (mm)	47.1 ± 3.5
IVS (mm)	9.3 ± 1.5
PD (mm)	8.8 ± 1.1
*e’* (cm/s)	7.09 ± 1.97
E/*e’*	11.24 ± 3.10
E/A	0.88 ± 0.29
LVEF (%)	63.87 ± 3.97

Data are reported as mean ± standard deviation for continuous variables and count/percentage for categorical variables

Abbreviations: *ACE* angiotensin converting enzyme, *LA* left atrium, *LVDD* left ventricular diastolic diameter, *IVS* inter-ventricular septum thickness, *PD* posterior wall thickness of left ventricle, *e'* mean mitral tissue velocity in early diastole, *E* mitral flow velocity in early diastole, *A* mitral flow velocity in late diastole, *LVEF* left ventricular ejection fraction

### LV diastolic function comparisons in HDL-C/hsCRP quartiles

Four quartile groups were separated according to HDL-C/hsCRP ratio. The HDL-C/hsCRP quartiles were Quartile 1 (HDL-C/hsCRP 0.0929–0.5288), Quartile 2 (HDL-C/hsCRP 0.5405–1.0119), Quartile 3 (HDL-C/hsCRP 1.0244–2.1667) and Quartile 4 (HDL-C/hsCRP 2.1739–9.2000). Comparisons of clinical and echocardiographic parameters in these quartile groups were shown in Table 2. Age, gender, smoking, blood pressure, blood glucose, blood cholesterol or medication use made no differences among these quartiles. However, the LV diastolic function indicated by E/e’ was significantly different. The lowest E/e’, indicating the best LV diastolic function, appeared in the highest HDL-C/hsCRP group.

**Table 2:** Comparisons of clinical and echocardiographic parameters in HDL-C/hsCRP quartile groups

	Quartile 1	Quartile 2	Quartile 3	Quartile 4	*P* value
Male gender (n/%)	22/43.1	21/41.2	20/39.2	21/41.2	0.983
Age (years)	61.0 ± 10.3	60.1 ± 12.0	59.3 ± 9.1	60.4 ± 9.6	0.861
Current smoker (n/%)	17/33.3	14/27.5	14/27.5	14/27.5	0.886
Statin use (n/%)	47/92.2	39/76.5	38/74.5	39/76.5	0.092
Other lipid regulator use (n/%)	9/17.6	6/11.8	7/13.7	8/15.7	0.854
ACE inhibitor use (n/%)	23/45.1	27/52.9	18/35.3	24/47.1	0.344
β blocker use (n/%)	28/54.9	24/47.1	25/49.0	26/51.0	0.876
SBp (mmHg)	142.6 ± 19.9	140.7 ± 20.7	141.9 ± 25.5	141.8 ± 25.3	0.981
DBp (mmHg)	84.8 ± 13.9	81.9 ± 16.7	81.7 ± 15.1	84.0 ± 15.5	0.674
BMI (kg/m^2^)	26.67 ± 2.93	25.92 ± 3.00	24.80 ± 2.33	23.32 ± 2.17	0.000
FPG (mmol/L)	6.15 ± 1.70	6.07 ± 1.93	5.43 ± 1.13	5.90 ± 2.48	0.215
HbA1C (%)	6.20 ± 0.97	6.34 ± 1.30	5.87 ± 1.23	5.94 ± 0.82	0.112
TC (mmol/L)	4.70 ± 1.21	4.93 ± 1.07	4.49 ± 0.87	4.87 ± 0.98	0.136
LDL-C (mmol/L)	3.07 ± 1.05	3.12 ± 0.86	2.90 ± 0.78	3.05 ± 0.87	0.625
TG (mmol/L)	2.04 ± 1.26	2.19 ± 2.12	1.56 ± 0.65	1.34 ± 1.10	0.006
UA (umol/L)	332.2 ± 71.9	311.1 ± 71.7	313.3 ± 94.6	282.2 ± 61.3	0.013
Cys C (mg/L)	0.86 ± 0.21	0.81 ± 0.18	0.83 ± 0.15	0.83 ± 0.24	0.684
LA (mm)	35.6 ± 2.9	36.1 ± 3.5	36.0 ± 4.2	34.4 ± 4.3	0.113
*e'* (cm/s)	6.9 ± 1.9	7.0 ± 1.9	6.8 ± 1.9	7.7 ± 2.1	0.090
E/*e'*	11.41 ± 2.91	11.98 ± 3.04	11.43 ± 3.36	10.14 ± 2.87	0.020
E/A	0.85 ± 0.31	0.93 ± 0.28	0.86 ± 0.29	0.87 ± 0.30	0.500
LVEF (%)	63.6 ± 4.2	63.6 ± 4.5	64.7 ± 3.6	63.6 ± 3.4	0.433

Data are reported as mean ± standard deviation for continuous variables and count/percentage for categorical variables

Abbreviations: *ACE* angiotensin converting enzyme, *SBp* systolic blood pressure, *DBp* diastolic blood pressure, *BMI* body mass index, *FPG* fasting plasma glucose, *HbA1C* hemoglobin A1C, *TC* total cholesterol, *LDL-C* low-density lipoprotein cholesterol, *TG* triglyceride, *UA* uric acid, *Cys C* cystatin C, *LA* left atrium, *e'* mean mitral tissue velocity in early diastole, *E* mitral flow velocity in early diastole, *A* mitral flow velocity in late diastole, *LVEF* left ventricular ejection fraction

### Correlations between HDL-C/hsCRP and *LV diastolic function*

In order to investigate if there was an association between HDL-C/hsCRP and LV diastolic function, we analyzed the correlations of different variables with E/*e’*. Of all the factors in Table 3, HDL-C/hsCRP was the most significant factor correlated with E/*e’*, making even stronger association with diastolic function than age, SBp, FPG, HDL-C or hsCRP. The correlation coefficient of HDL-C/hsCRP was -0.232 (*P* < 0.001) and it demonstrated a negative correlation between HDL-C/hsCRP ratio and E/*e’*. HDL-C/hsCRP was also the most significant independent determinant of E/*e’* in multiple regression analysis when adjusted by all the significant variables in univariate linear correlations, including age, SBp, FPG, Cys C and hsCRP. The standardized correlation coefficient β was -0.258 (*P* < 0.0005) (Table 4).

**Table 3:** Univariate linear correlations between E/*e’* and different variables

	r	*P* value
Male gender	−0.053	0.450
Age	0.173	0.013
Smoking	−0.001	0.983
Heart rate	−0.136	0.052
Systolic blood pressure	0.185	0.008
Diastolic blood pressure	0.067	0.344
Body mass index	0.123	0.079
Fasting plasma glucose	0.188	0.007
Hemoglobin A1C	0.138	0.052
Uric acid	0.017	0.817
Total cholesterol	0.001	0.986
Low-density lipoprotein cholesterol	0.022	0.760
High-density lipoprotein cholesterol	−0.126	0.072
Triglyceride	0.053	0.451
Cystatin C	0.152	0.044
High-sensitive C-reactive protein	0.142	0.042
HDL-C/ hsCRP	−0.232	< 0.001

Abbreviations: *HDL/ hsCRP* ratio of high-density lipoprotein cholesterol to high-sensitive C-reactive protein

**Table 4:** Multiple Regression Analysis for relevant parameters and E/*e’*

	Standardized β coefficient	P value
Age	0.181	< 0.05
Systolic blood pressure	0.143	< 0.05
Fasting plasma glucose	0.149	< 0.05
HDL-C/ hsCRP	−0.258	< 0.0005

Significant variables in univariate linear correlations are included in this multivariate model. Only independent parameters are shown

Abbreviations: *HDL/ hsCRP* ratio of high-density lipoprotein cholesterol to high-sensitive C-reactive protein

### The ablility of HDL-C/hsCRP in predicting LV diastolic dysfunction

**Fig. 1:**
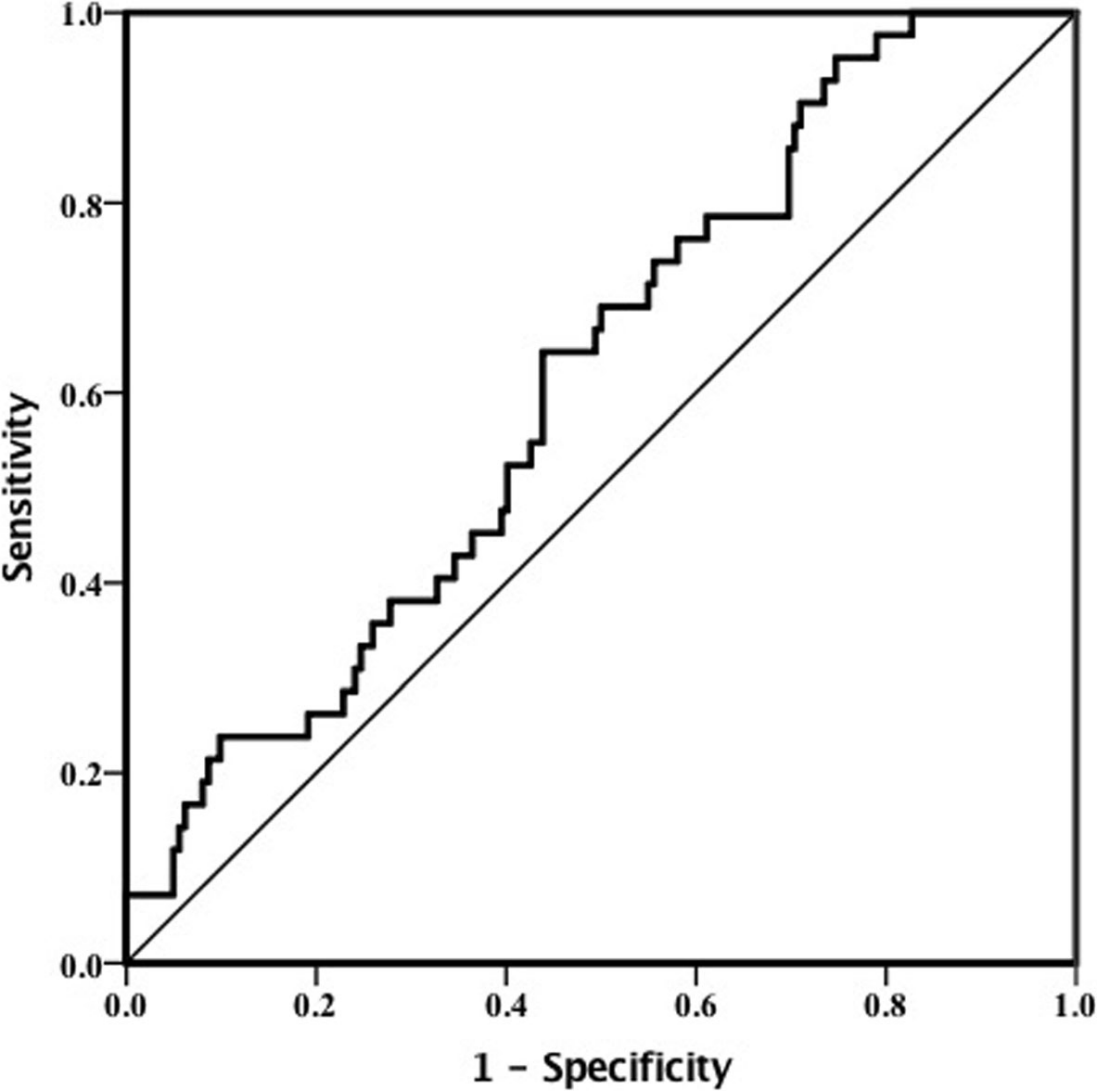
Receiver operating characteristic curve of HDL-C/hsCRP in predicting left ventricular diastolic dysfunction E/e’ > 14.

As mentioned above, LV diastolic dysfunction was defined as E/*e’* > 14. In logistic regression analysis, HDL-C/hsCRP was proved to be a protective factor of LV diastolic dysfunction (OR = 0.649, 95%CI 0.444–0.948, *P* = 0.025) (Table 5). Receiver operating characteristic (ROC) curve was plotted to test the ability of HDL-C/hsCRP in predicting LV diastolic dysfunction (Fig. 1). The sensitivity of using HDL-C/hsCRP < 0.98 to indicate LV diastolic dysfunction was 64.3% and the specificity was 56.2%. The area under the curve (AUC) was 0.613.

**Table 5:** Logistic Regression Analysis for association between relevant parameters and LV diastolic dysfunction

	OR	95%CI	*P* value
Age	1.048	1.005–1.093	0.028
Systolic blood pressure	1.018	1.001–1.036	0.034
HDL-C/ hsCRP	0.649	0.444–0.948	0.025

Variables included are significant parameters in univariate linear correlations. Only significant parameters are shown

Abbreviations: *CI* confidence interval, *OR* odd ratio, *HDL/ hsCRP* ratio of high-density lipoprotein cholesterol to high-sensitive C-reactive protein

### Relationship between HDL-C/hsCRP and CV risk factors

**Fig. 2:**
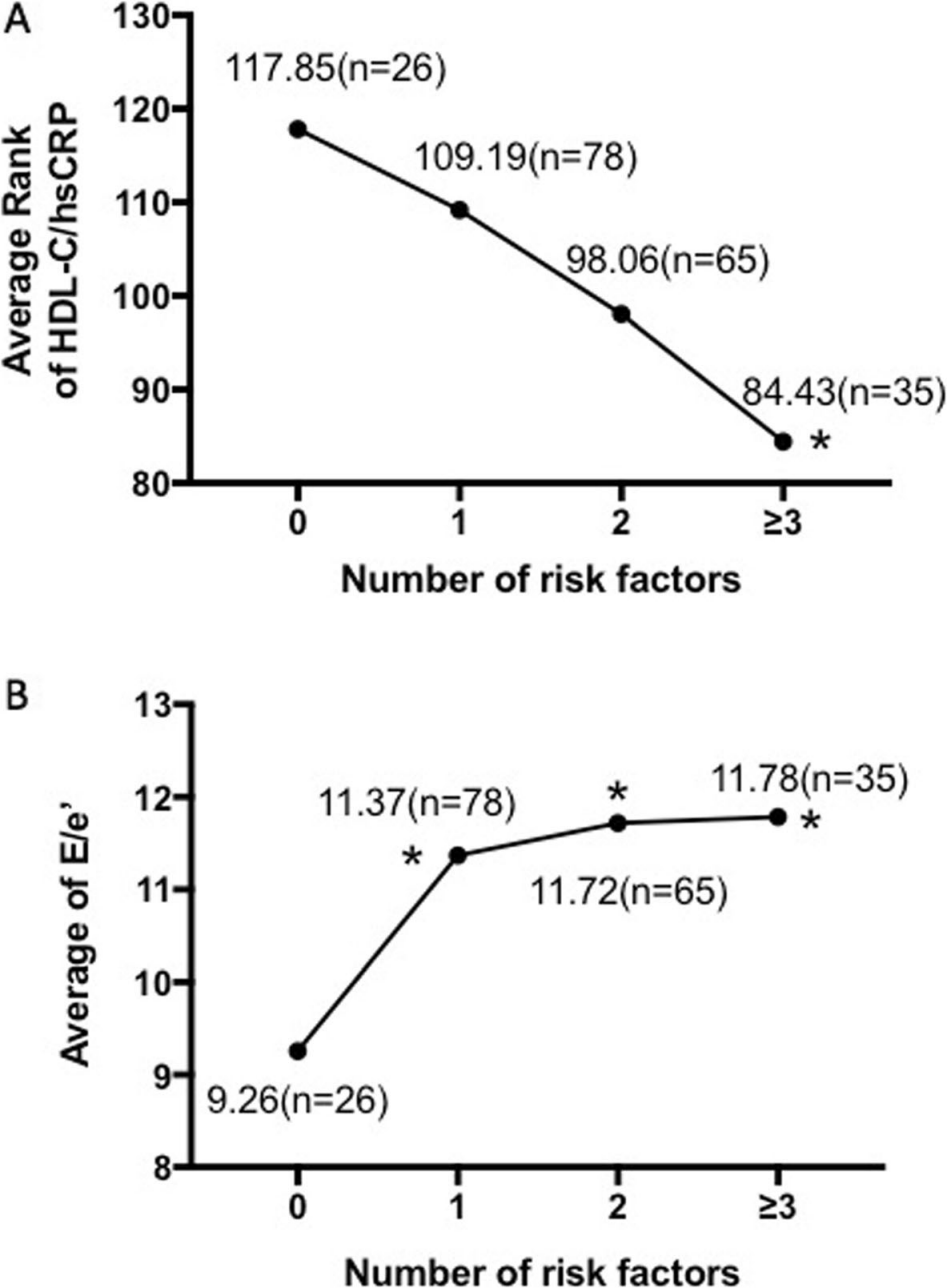
Trends of HDL-C/hsCRP and LV diastolic function with increased CV risk factors. **a** Decreased trend of HDL-C/hsCRP with increased CV risk factors. HDL-C/hsCRP was ranked from the lowest value to the highest value. **b** Increased trend of E/e’ with increased CV risk factors. The asterisk (*) represents a significant difference from those without CV risks (*P* < 0.05)

Number of CV risk factors (current smoking, hypertension, diabetes, obese and hypercholesterolemia) was counted in each subject. HDL-C/hsCRP ratio was ranked from the lowest value to the highest value. Average ranks were compared according to the number of CV risk factors. As the risk factors increased, HDL-C/hsCRP presented a reduced tendency. HDL-C/hsCRP ratio in subjects with 3 or more CV risk factors was significantly lower compared with those without CV risks (Fig. 2a). At the same time, E/*e*’ presented an increasing trend with increased rate of CV risk factors (Fig. 2b).

## Discussion

This study contributed three new findings concerning HDL-C, hsCRP and LV diastolic function. First, we demonstrated a negative association of HDL-C/hsCRP and echocardiographic parameter E/*e’,* indicating a positive association of HDL-C/hsCRP and LV diastolic function. Second, HDL-C/hsCRP < 0.98 could be used for predicting LV diastolic dysfunction with 64.3% sensitivity and 56.2% specificity. Last, HDL-C/hsCRP and LV diastolic function both varied with the quantity of CV risk factors. Those with more CV risk factors tended to show lower HDL-C/hsCRP and worse LV diastolic function.

Previously, Masugata et al found that there was a relationship between hsCRP and LV diastolic function in patients with cardiovascular risk factors regardless of coronary plaque and elevated hsCRP meant reduced LV diastolic function rather than LV hypertrophy [20]. It is also reported that in treated essential hypertensive patients HDL-C is favorably associated with LV diastolic function [21]. In addition, Manabu and his colleagues proved that a combination of CRP and HDL-C might predict long-term outcomes in patients with CAD under statin therapy after percutaneous coronary intervention [22]. In this study, we combined HDL-C and hsCRP and found HDL-C/hsCRP ratio strongly correlated with LV diastolic function in subjects without significant coronary plaques. The absolute value of the correlation coefficient of HDL-C/hsCRP was higher than either HDL-C or hsCRP in univariate correlation, and only HDL-C/hsCRP, rather than HDL-C or hsCRP, was independent in multiple regression. These results reflected superiority of HDL-C/hsCRP to either HDL-C or hsCRP when correlating with LV diastolic function. According to the logistic regression analysis, HDL-C/hsCRP ratio was a protective marker of diastolic dysfunction. It implicated that high HDL-C/hsCRP was not likely to be with LV diastolic dysfunction and low HDL-C/hsCRP ratio might help recognize LV diastolic dysfunction E/*e* > 14. Therefore, we set a value of HDL-C/hsCRP< 0.98 for predicting E/e’ > 14 with 61.3% accuracy.

HDL is a group of heterogeneous particles with pleiotropic beneficial effects originating from its complicated structure and ingredients [23]. Anti-inflammation character is one of its main functions besides well-known cholesterol reverse transport [24]. HDL and its mimetic peptide improved diastolic function in low-density lipoprotein receptor deficient (LDLr(−/−)) mice and cholesterol-fed rabbits respectively [25, 26].However, inflammation leads to impaired anti-inflammatory capacity of HDL and promotes transformation of HDL into pro-inflammatory particles [27].Impaired anti-inflammatory property of HDL was associated with heart failure [28]. Although HDL-C refers to the cholesterol content in HDL, not HDL particle itself, it reflects plasma HDL anti-inflammation ability to some extent because in inflammatory conditions, HDL-C decreases with impaired cholesterol efflux and reduced anti-inflammation ability of HDL as well [29–32]. As a result, the combination of HDL-C and hsCRP actually improved the detection ability of systemic inflammatory state. In this study, HDL-C/hsCRP was found to decrease with increased rate of CV risks, which could induce chronic inflammation. It is rational that more risk factors mean severer systemic inflammatory status and worse resistance to inflammation. Single CV risk factor only affects part of LV diastolic function, but HDL-C/hsCRP ratio seems to be a comprehensive indicator for all the CV risk factors.

Aging, female gender, hypertension, obesity and diabetes are the common reasons of LV diastolic dysfunction [33]. CAD with obvious plaque is also a leading reason for LV diastolic function impairment [18]. Because LV diastolic dysfunction and CAD share similar risk factors, we excluded subjects with coronary stenosis ≥50%, which would probably impact the usefulness of HDL-C/hsCRP ratio in LV diastolic function evaluation. Although atrial fibrillation is another possible reason causing LV diastolic function impairment especially among the patients with fast heart rate, we also excluded this kind of patients in this study, since atrial fibrillation affects the accuracy of diastolic function measurement [34].Because this was a cross-section research with relative small sample of subjects instead of a cohort from population, we did not stratify different groups of age, which had impacts on LV diastolic function. That was why we choose E/e’, a less age-dependent parameter, as indicator of LV diastolic function. Similarly, we did not stratify medications such as ACE inhibitor and β blocker, which might also impact LV diastolic function. Instead, we compared the percentages of ACE inhibitor and β blockers used in different HDL-C/hsCRP quartiles and there were no significant differences. Nevertheless, this did not influence differentiating LV diastolic function change. We noticed both HDL-C and hsCRP could be adjusted by statins, but we did not exclude those who were using statins, because we wanted to perform the investigation in a general situation where statins were commonly used for the prevention of cardiovascular diseases. Since statins were also reported to improve diastolic cardiac function [35], it is likely that the link between HDL-C/hsCRP ratio and LV diastolic function exists regardless of medication.

There are some limitations of this study. First, the correlation strength between HDL-C/hsCRP and LV diastolic function needed to be proved by a larger stratified sample. Second, the subjects were all free from heart failure symptoms. As a result, it was still not clear whether HDL-C/hsCRP could be used in the evaluation of diastolic function in heart failure patients. Further investigations are needed for complete explanations.

## Conclusions

In summary, HDL-C/hsCRP ratio, a comprehensive indicator reflecting systemic inflammation status, closely correlates with LV diastolic function in absence of significant coronary atherosclerosis. Low HDL-C/hsCRP ratio tends to relate with LV diastolic dysfunction.

## Data Availability

All data generated and analysed during this study are included in this published article.

## Abbreviations

A: Mitral flow velocity in late diastole
ACE: Angiotensin converting enzyme
AUC: Area under the curve
BMI: Body mass index
CAD: Coronary atherosclerotic disease
CI: Confidence interval
CV: Cardiovascular
CysC: Cystatin C
DBp: Diastolic blood pressure
e': Mean mitral tissue velocity in early diastole
E: Mitral flow velocity in early diastole
FPG: Fasting plasma glucose
HbA1C: Hemoglobin A1C
HDL-C: High-density lipoprotein cholesterol
hsCRP: High-sensitive C-reactive protein
IVS: Inter-ventricular septum thickness
LA: Left atrium
LDL-C: Low-density lipoprotein cholesterol
LV: Left ventricular
LVDD: Left ventricular diastolic diameter
LVEF: Left ventricular ejection fraction
OR: Odd ratio
PD: Posterior wall thickness of left ventricle
ROC: Receiver operating characteristic
SBp: Systolic blood pressure
SD: Standard deviation
TC: Total cholesterol
TG: Triglyceride
UA: Uric acid
